# Immunogenicity and safety of an inactivated enterovirus 71 vaccine coadministered with trivalent split-virion inactivated influenza vaccine: A phase 4, multicenter, randomized, controlled trial in China

**DOI:** 10.3389/fimmu.2022.1080408

**Published:** 2022-12-08

**Authors:** Yaping Chen, Yanhui Xiao, Ying Ye, Feng Jiang, Hanqing He, Linyun Luo, Haiping Chen, Lubin Shi, Qiuyue Mu, Wei Chen, Xue Guo, Min Zhang, Jun Li, Qinghu Guan, Zhiping Chen, Xiaoming Yang

**Affiliations:** ^1^ Immunization Programme Department, Zhejiang Provincial Center for Disease Control and Prevention, Hangzhou, Zhejiang, China; ^2^ Medical Affairs Department, China National Biotec Group Company Limited, Beijing, China; ^3^ Institute for Communicable Disease Control and Prevention, Henan Provincial Center for Disease Control and Prevention, Zhengzhou, Henan, China; ^4^ Institute of Expanded Programme on Immunization, Guizhou Provincial Center for Disease Control and Prevention, Guiyang, Guizhou, China; ^5^ Institute of Expanded Programme on Immunization, Henan Provincial Center for Disease Control and Prevention, Zhengzhou, Henan, China; ^6^ Medical Affairs Department, Wuhan Institute of Biological Products Company Limited, Wuhan, Hubei, China; ^7^ Medical Affairs Department, Changchun Institute of Biological Products Company Limited, Changchun, Jilin, China; ^8^ Research and Development Department, National Engineering Technology Research Center for Combined Vaccines, Wuhan Institute of Biological Products Company Limited, Wuhan, Hubei, China

**Keywords:** coadministration, inactivated enterovirus 71 vaccine, trivalent split-virion inactivated influenza vaccine, immunogenicity, safety

## Abstract

**Background:**

Few data exist on the immunogenicity and safety of an inactivated enterovirus 71 vaccine (EV71 vaccine) coadministered with trivalent split-virion inactivated influenza vaccine (IIV3) in infants.

**Methods:**

This trial was a phase 4, randomized, controlled trial. Infants aged 6-11 months were eligible, with no history of hand, foot and mouth disease (HFMD) and no history of EV71 vaccine or any influenza vaccine. Eligible infants were randomly assigned to EV71+IIV3 group, EV71 group or IIV3 group. Blood samples were collected on day 0 and 56.

**Results:**

Between September 2019 and June 2020, 1151 infants met eligibility criteria and 1134 infants were enrolled. 1045 infants were included in the per-protocol population, including 347 in the EV71+IIV3 group, 343 in the EV71 group, and 355 in the IIV3 group. The seroconversion rate (98.56% vs 98.54%; seroconversion rates difference of 0.02% [95% CI: 0.70-0.98]) and GMT (419.05 vs 503.72; GMT ratio of 0.83 [95% CI 0.70 - 0.98]) of EV71 neutralizing antibodies in the EV71+IIV3 group was not inferior to those in the EV71 group. The non-inferiority results for influenza virus antibodies (A/H1N1, A/H3N2 and B) showed that the seroconversion rates and GMTs of the EV71+IIV3 group were non-inferiority to those of the IIV3 group. Systemic and local adverse event rates were similar between groups. None of serious adverse events (SAEs) were related to vaccination.

**Conclusions:**

Coadministration of the EV71 vaccine with IIV3 was safe and did not interfere with immunogenicity. These findings support a viable immunization strategy for infants with the EV71 vaccine coadministered with IIV3 in China.

This trial is registered with ClinicalTrials.gov, number NCT04091880.

## Introduction

One of the major pathogens of hand, foot and mouth disease (HFMD) in children under the age of five is enterovirus type 71 (EV71) (1, 2). Infection with EV71 can cause severe HFMD with neurological complications such as meningitis and pulmonary edema (3, 4). More than 90% of HFMD deaths are associated with EV71 in China from 2008 to 2012 (5). The seasonal influenza virus is a leading contributor of acute lower respiratory infection (ALRI), accounting for three to five million symptomatic cases and 290 to 645 thousand deaths per year, globally (6). As a high‐risk population, young children can experience severe influenza‐related morbidity and mortality (7, 8). In 2018, there were an estimated 870 thousand influenza-virus-associated ALRI hospital admissions and up to 34800 overall influenza-virus-associated ALRI deaths in children under the age of 5 worldwide (7).

The incidence rate of HFMD and influenza decreased significantly during the COVID-19 pandemic in 2020 as a result of non-pharmaceutical interventions implemented, but rebound sharply after the relaxation of comprehensive intervention policies in China beginning in 2021 (9–11). Since May 2022, the intensity of influenza activity in southern China, has entered the summer peak period, reaching the highest level compared with the same period of five years, with A (H3N2) subtype as the absolute dominant strain. Therefore, in the context of COVID-19 pandemic, HFMD and influenza remain major public health threats.

Both influenza vaccine and EV71 vaccine have been found to be the most cost-effective measure for disease control in children (12, 13). The EV71 vaccine developed by Wuhan Institute of Biological Products has been approved and is available in China for use in children aged 6 months to 36 months with a schedule of two doses separated by 1 month. Two doses of trivalent split-virion inactivated influenza vaccine (IIV3, 0.25 mL/dose) with an interval of 4 weeks, are recommended for children aged 6 months to 36 months in China. Given the decline in childhood immunization coverage rates during the COVID-19 pandemic (14), coadministration of EV71 vaccine and IIV3 for children aged 6 months or older could provide protection against HFMD and influenza while also improving the coverage of both vaccines and reducing clinic visits. Therefore, the immunogenicity and safety of the EV71 vaccine coadministered with IIV3 versus EV71 or IIV3 administration alone needs to be evaluate.

## Methods

### Study design

A phase 4, multicenter, randomized, controlled trial was conducted in three centers in Liandu District in Lishui City, Zhejiang Province; Boai County in Jiaozuo City, Henan Province and Qianxi County in Bijie City, Guizhou Province in China. Informed consent was obtained signed by a parent or legal guardian of the participant before receiving any study treatment. The study protocol was approved by the ethical review committee of Zhejiang provincial center for disease control and prevention (CDC) (T-043-R), Henan provincial CDC (2019-KY-007-02) and Guizhou provincial CDC (2019–0001). This trial is registered with ClinicalTrials.gov, number NCT04091880.

### Participants

The participants were recruited in community by investigators from Liandu District CDC, Boai County CDC and Qianxi County CDC. Infants were eligible if they met the following criteria: 6-11 months of age, ≥14 days since last vaccination, no history of EV71 vaccine or influenza vaccine, no history of HFMD, and body temperature of ≤37.0°C. Infants were excluded from the first dose if they met one of the following criteria: a history of allergy to vaccines, suffered convulsions, epilepsy, cerebral disease, or mental disease, hypoimmunity or the receipt of non-specific immunoglobulin therapy in the past 3 months. Infants who developed severe allergies or adverse events related to the study vaccination, as well as any new events were excluded from the second dose.

### Vaccines

The EV71 vaccine (0.5 mL/dose, lot number 201810076, Wuhan, China) was manufactured by the Wuhan Institute of Biological Products and contained ≥ 3.0 EU of antigen with a seed virus of EV71 strain AHFY087VP5 (genotype C4) (15). The EV71 vaccine was approved for use in China with a schedule of two doses, 1 month apart, for children aged 6 months to 36 months. The IIV3 (0.25 mL/dose, lot number T20190701, Changchun) was manufactured by the Changchun Institute of Biological Products. The hemagglutinins used in IIV3 were A/Brisbane/02/2018(H1N1) pdm09 -like virus, A/Kansas/14/2017(H3N2) -like virus and B/Colorado/06/2017 -like virus (B/Victoria/2/87 lineage). The IIV3 was approved a two-dose schedule with an interval of 2-4 weeks for children aged 6 months to 3 years.

### Randomization and masking

Random number was generated by an independent statistician using block randomization by SAS 9.4 (block size:9). All eligible infants were assigned a unique random number by investigators *via* a separate envelope that was used to identify all interventions. According to the random number, infants were randomly allocated to the EV71+IIV3 group (EV71 vaccine coadministered with IIV3), the EV71 group (EV71 vaccine administered alone) and the IIV3 group (IIV3 vaccine administered alone) at a ratio of 1:1:1. An open-label trial was conducted to reduce unnecessary injection. For the study group assignment, infants, parents and investigators were not masked, but laboratory technicians were.

### Procedures

Following enrolment, study physicians obtained the baseline demographic of participant to complete a uniform questionnaire.

The EV71+IIV3 group received the first dose of EV71 vaccine and the first dose of IIV3 on day 0, and received the second dose of EV71 vaccine and the second dose of IIV3 on day 28. The EV71 group received two doses of EV71 vaccine on day 0 and day 28, respectively. The IIV3 group received two doses of IIV3 on day 0 and day 28, respectively. All participants were allowed to complete their second vaccination within a predetermined window of 7 days. Blood samples of 3 mL were collected before the first vaccination (day 0) and 28 days after completion of the second vaccination (day 56). The second blood sample could be collected within a predetermined window of 15 days.

### Immunogenicity assessment

Neutralizing antibody titers against EV71 were measured by the Chinese National Institute for Food and Drug Control using AHFY087VP5 strain based cytopathic effect inhibition assay (15). Seropositive for EV71 was defined as an antibody titer of ≥1:8. EV71 seroconversion was defined as an antibody titer of ≥1:8 if baseline antibody titer of <1:8, or a minimum 4-fold increase if baseline antibody titer of ≥1:8. The Chinese National Institute for Food and Drug Control used a hemagglutination inhibition test (details are in the **Supplementary Material**) on the IIV3 antibody titers. Seropositivity for IIV3 antibodies was defined as a titer of ≥1:40 for influenza antibodies. The IIV3 antibody seroconversion was defined as a titer of ≥1:40 if the pre-vaccination antibody titer < 1:10, or at least a 4-fold increase if the pre-vaccination antibody titer of ≥1:10.

### Safety assessment

All participants were monitored by the study physician for 30 minutes after vaccination for adverse events (AEs). Any systemic or local AEs after vaccinations were recorded using a diary card by the parents or legal guardians of the participants that occurred within 28 days. Serious adverse events (SAE) within 6 months after the first vaccination were collected by the investigators using a Chinese surveillance system on adverse events following immunization (AEFI). Systemic solicited AEs included fever, rash, diarrhea, vomiting, decreased appetite, irritability and drowsiness. Local solicited AEs included pain, redness, induration, swelling and pruritus. It was assumed that a solicited AE occurring within 7 days of vaccination was related to the vaccine.

### Outcomes

The primary outcome was the non-inferiority of the seroconversion rate and geometric mean titer (GMT) between the EV71+IIV3 group and the EV71 group and influenza virus between the EV71+IIV3 group and the IIV3 group. The secondary outcome was the rate of AEs within 28 days of each immunization.

### Statistical analysis

PASS version 11 was used to estimate the sample size. Assuming the seroconversion rates for EV71 and influenza virus in the EV71+IIV3 group were non-inferior to those in the EV71 group or IIV3 group. We estimated a minimum sample size of 366 in each group, a one-sided α of 0.025, a power of 80%, a non-inferiority margin of –10%, a lowest seroconversion rate of 70% and potential loss to follow-up of 15%. Considering the multicenter design and block size, a sample size of 378 per group was determined, and total sample size of the three groups was 1134.

STATA 17 was used for statistical analyses. The baseline characteristics were reported as mean ± standard deviation (SD) for continuous variables and n (%) for categorical variables. Nonparametric test was used to compare the age of participants between three groups and χ2 test was used to compare the proportion of males between three groups. The immunogenicity was assessed in the per-protocol population. Half of the lower detection limit was calculated an antibody titer below the detection limit (lower limit of detection of EV71 antibody titer was 1:8, lower limit of detection of influenza virus was 1:10). 2-fold of the upper detection limit was calculated an antibody titer above the detection limit (upper limit of detection of EV71 antibody titer was 1:16384, upper limit of detection of influenza virus was 1:5120). The Clopper-Pearson method was used to calculated two-sided 95% confidence intervals (CIs). If the lower limit of the 95% CI of the seroconversion rate difference between groups was ≥–10%, the EV71+IIV3 group was determined to be non-inferior to the EV71 group and the IIV3 group. The antibody titers were log10 transformed and present them as GMTs with 95% CIs and geometric mean increases (GMIs) with 95% CIs. If the lower limit of 95% CI of GMT ratio was ≥0.67, the EV71+IIV3 group was non-inferior to the EV71 group and the IIV3 group. The safety outcome was evaluated in infants who received at least one dose of vaccine, and it was presented descriptively as n (%) for systemic and local AEs. Fisher’s exact test and χ2 test were used to compare the rates between groups. The GMTs and GMIs were compared between groups using a nonparametric test and a Student’s t-test. The comparison was significance if the two-sided p value was <0.05.

## Results

**Figure 1:**
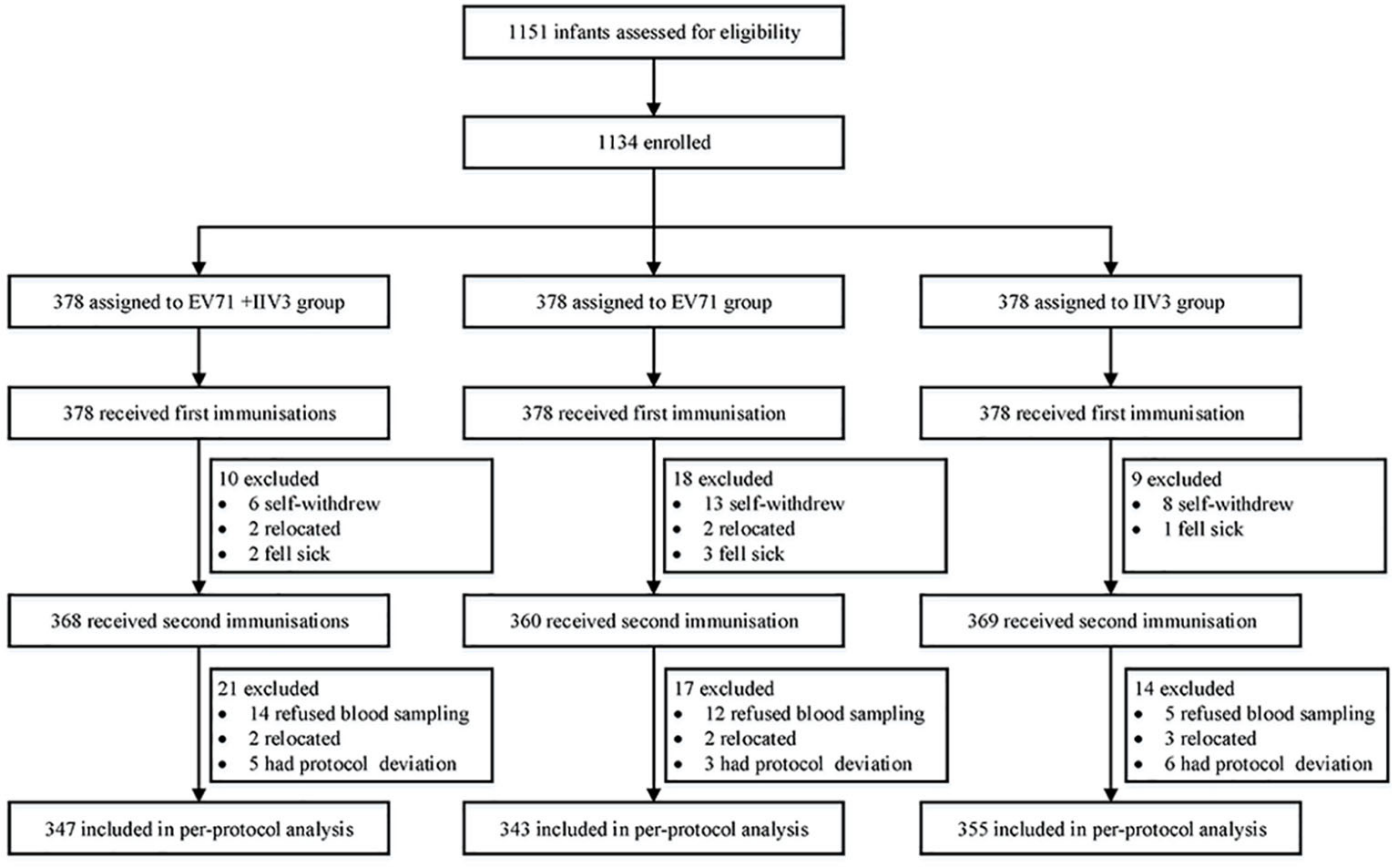
Trial profile. EV71= inactivated enterovirus 71 vaccine. IIV3= trivalent split-virion inactivated influenza vaccine.

Between September 2019 and June 2020, a total of 1134 infants from three research centers were enrolled and randomly assigned to the EV71+IIV3 group (n =378), the EV71 group (n =378) and the IIV3 group (n =378). All participants completed blood sampling and vaccination on day 0. In total, 1045 infants were included in the per-protocol population (**Figure 1**). The mean age of the EV71+IIV3 group, the EV71 group and the IIV3 group were 8.49 months, 8.48 months and 8.54 months and the proportion of males was 46.15% (174/347), 45.89% (173/343) and 44.83% (169/355), respectively. In each group, the age and sex of the participants were comparable (**Table 1**).

**Table 1:** Baseline characteristics in the per-protocol population.

	EV71+IIV3 group (n=347)	EV71 group (n=343)	IIV3 group (n=355)	P
**Age (months)**				
Mean ± SD	8.49 ± 0.09	8.48 ± 0.09	8.54 ± 0.08	0.948
**Sex**				
Male, n (%)	174 (46.15)	173 (45.89)	169 (44.83)	0.711

**Figure 2:**
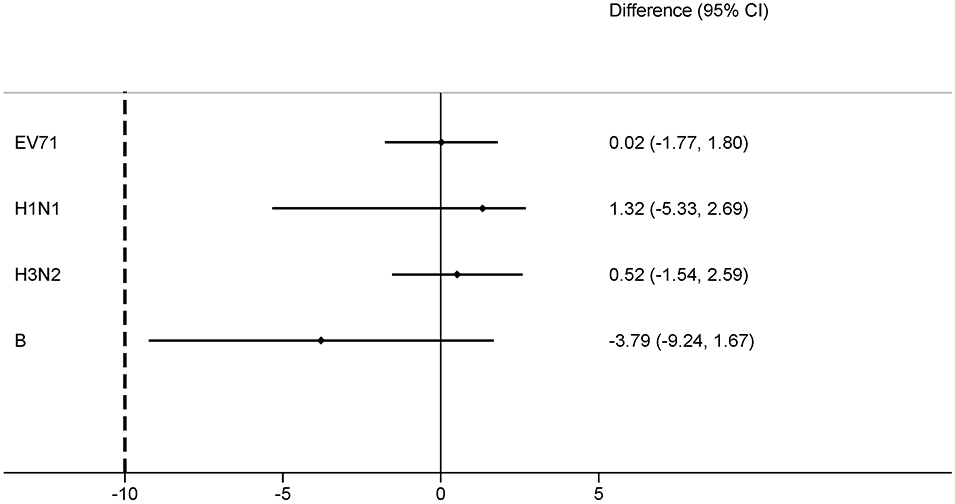
Differences in the proportion of seroconversion rates for EV71+IIV3 group versus EV71 group or IIV3 group. The seroconversion rate differences were measured between the EV71+IIV3 group and the EV71 group or IIV3 group with two-sided 95% CIs. For each type antibody, the EV71+IIV3 group was non-inferior to EV71 group or IIV3 group for the seroconversion rates (the lower limit of the 95% CI of the seroconversion rate difference between groups was ≥–10%).

**Figure 3:**
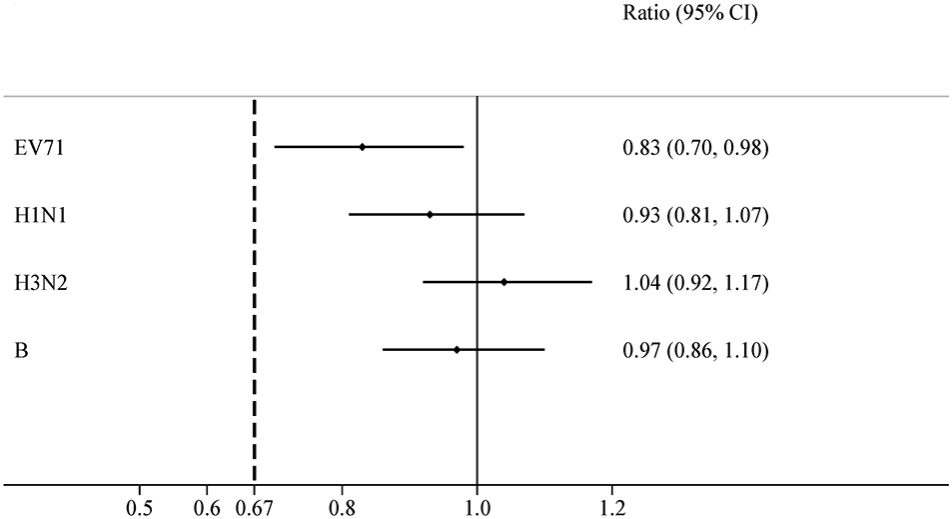
GMT ratios for EV71+IIV3 group versus EV71 group or IIV3 group. The GMT ratios were measured between the EV71+IIV3 group and the EV71 group or IIV3 group with two-sided 95% CIs. For each type antibody, the EV71+IIV3 group was non-inferior to EV71 group or IIV3 group for GMT ratios (the lower limit of 95% CI of GMT ratio between groups was ≥0.67).

For the EV71+IIV3 group and EV71 group, the seroconversion rates of EV71 neutralizing antibodies were 98.56% (342/347) and 98.54% (338/343) on day 56. The seroconversion rates difference of 0.02% (95% CI: –1.77-1.80) indicated the non-inferiority. The GMT of the EV71 neutralizing antibodies was 419.05 (95% CI: 372.55–471.35) in the EV71+IIV3 group and 503.72 (95% CI: 447.99–566.39) in the EV71 group on day 56, the GMT was slightly lower in the EV71+IIV3 group than in the EV71 group (P=0.030). However, non-inferiority was met for the GMT of EV71 neutralizing antibodies, and the GMT ratio was 0.83 (95%CI: 0.70–0.98). (**Table 2**; **Figures 2**, **3**)

**Table 2:** Antibody responses to EV71, A/H1N1, A/H3N2 and B in the per-protocol population.

	EV71+IIV3 group	EV71 group or IIV3 group	P
**EV71**	n=347	n=343	
**Pre-vaccination**			
Seropositive, n (%)	88(25.36)	92(26.82)	0.662
GMT (95%CI)	5.34(4.93, 5.77)	5.52(5.05, 6.04)	0.753
**Post-vaccination**			
Seropositive, n (%)	347(100)	343(100)	1.000
Seroconversion, n (%)	342(98.56)	338(98.54)	0.662
GMT (95%CI)	419.05(372.55, 471.35)	503.72(447.99, 566.39)	0.030
GMI (95%CI)	78.52(69.23, 89.05)	91.23(79.69, 104.44)	0.053
**H1N1**	n=347	n=355	
**Pre-vaccination**			
Seropositive, n (%)	17(4.90)	16(4.51)	0.806
GMT (95%CI)	5.91(5.49, 6.37)	5.91(5.48, 6.38)	0.832
**Post-vaccination**			
Seropositive, n (%)	320(92.22)	335(94.37)	0.255
Seroconversion, n (%)	317(91.35)	329(92.68)	0.518
GMT (95%CI)	119.76(108.37, 132.36)	128.57(116.99, 141.30)	0.366
GMI (95%CI)	20.25(18.32, 22.39)	21.74(19.76, 23.92)	0.315
**H3N2**	n=347	n=355	
**Pre-vaccination**			
Seropositive, n (%)	5(1.44)	6 (1.69)	0.790
GMT (95%CI)	5.54 (5.31, 5.77)	5.72 (5.47, 5.98)	0.110
** Post-vaccination**			
Seropositive, n (%)	342(98.56)	348(98.03)	0.587
Seroconversion, n (%)	341(98.27)	347(97.75)	0.619
GMT (95%CI)	148.60(136.61, 161.65)	143.43(131.37, 156.59)	0.351
GMI (95%CI)	26.84(24.80, 29.05)	25.07(23.19, 27.10)	0.227
**B**	n=347	n=355	
**Pre-vaccination**			
Seropositive, n (%)	0 (0)	0 (0)	–
GMT (95%CI)	5.08 (5.02, 5.14)	5.09 (5.02, 5.15)	0.829
**Post-vaccination**			
Seropositive, n (%)	285(82.13)	304(85.63)	0.207
Seroconversion, n (%)	284(81.84)	304(85.63)	0.173
GMT (95%CI)	59.76(54.77, 65.21)	61.70(56.49, 67.40)	0.613
GMI (95%CI)	11.76(10.77, 12.84)	12.13(11.11, 13.23)	0.630

On day 56, similar the seroconversion rates were observed between the EV71+IIV3 group and the IIV3 group for A/H1N1 (91.35% [317/347] vs 92.68% [329/355]), A/H3N2 (98.27% [341/347] vs 97.75% [347/355]) and B (81.84% [284/347] vs 85.63% [304/355]). The EV71+IIV3 group was non-inferior to the IIV3 group for seroconversion rates of A/H1N1 (the seroconversion rates differences: 1.32% [95%CI: -5.33–2.69]), A/H3N2 (0.52% [95%CI: -1.54–2.59]) and B (-3.79% [95%CI: -9.24–1.67]). For the EV71+IIV3 group and the IIV3 group, the GMTs of antibodies against A/H1N1 were 119.76 and 128.57, A/H3N2 were 148.60 and 143.43, and B were 59.76 and 61.70. The GMT ratio was 0.93 [95%CI: 0.81–1.07] for A/H1N1, 1.04 [95%CI:0.92–1.17] for A/H3N2, 0.97 [95%CI:0.86–1.10] for B, which lower limits of both the CIs above the non-inferiority margin. (**Table 2**; **Figures 2**, **3**)

Any AEs within 28 days after vaccination occurred in 119 (31.48%) of 378 participants in the EV71+IIV3 group, 100 (26.46%) of 378 participants in the EV71 group and 116 (30.69%) of 378 in the IIV3 group. There was no significant difference in AEs rates between the three groups (P>0.05). The most common AE was fever. (**Table 3**) Similar results were observed for vaccine-related AEs that occurred within 7 days of vaccination. (**Supplementary Material**) A total of four cases of SAEs were reported: two in the EV71+IIV3 group and two in the EV71 group, all of which were not related to vaccination.

**Table 3:** Reported adverse events following any vaccination within 28 days.

Adverse events	EV71+IIV3 group(n=378)	EV71 group(n=378)	IIV3 group(n=378)	P
**Total, n (%)**	119(31.48)	100(26.46)	116(30.69)	0.266
**Systemic, n (%)**	116(30.69)	98(25.93)	113(29.89)	0.302
Fever, n (%)	71(18.78)	60(15.87)	54(14.29)	0.237
Rash, n (%)	12(3.17)	9(2.38)	7(1.85)	0.499
Diarrhea, n (%)	10(2.65)	5(1.32)	10(2.65)	0.360
Vomiting, n (%)	4(1.05)	2(0.53)	2(0.53)	0.604
Decreased appetite, n (%)	1(0.26)	0(0)	0(0)	1.000
Irritability, n (%)	0(0)	0(0)	1(0.26)	1.000
Drowsiness, n (%)	1(0.26)	0(0)	0(0)	1.000
Unsolicited, n (%)	44(11.64)	46(12.17)	56(14.81)	0.377
**Local, n (%)**	5(1.32)	3(0.79)	6(1.59)	0.603
Redness, n (%)	0(0)	2(0.53)	0(0)	0.333
Induration, n (%)	4(1.06)	3(0.79)	6(1.59)	0.526
Unsolicited, n (%)	1(0.26)	0(0)	1(0.26)	1.000

## Discussion

In this phase 4, multicenter, randomized, controlled trial, we found that the coadministration of EV71 vaccine with IIV3 in immunogenicity against EV71 and influenza virus (H1N1, H3N2, and B) was non-inferior to two doses of EV71 vaccine or IIV3 given 28 days apart. In infants aged 6-11 months who received EV71 vaccine and IIV3 simultaneously or separately, seroconversion rates of EV71, H1N1, H3N2 and B were above 98%, 90%, 97% and 80%, respectively. The high seroconversion rate and good safety data supported the coadministration of EV71 vaccine with IIV3.

The EV71 vaccine has been approved and widely used in China, showing good immunogenicity, safety and efficacy. In a randomized, controlled trial involving 10007 healthy infants and young children aged 6 to 35 months, the EV71 neutralizing antibody response (≥1:8) rate was 98.8% with two-dose vaccination, and the frequencies of AEs were similar in the EV71 vaccine group and placebo group (16). A follow-up study showed that two doses of EV71 vaccine provided 94.84% efficacy in preventing EV71-associated HFMD after 2 years in children aged 6-35 months (17). A real-world study conducted in Chengdu city reported that the actual average incidence rate of EV71-associated HFMD in 2017–2018 was estimated to be 60% lower than predicted incidence rate when EV71 vaccine was not approved for marketing in China (2011–2017), and the number of severe HFMD cases was 52% fewer than predicted (18).

There is currently no published data on the immunogenicity and safety of coadministration of EV71 vaccine with IIV3. A multicenter, phase 4 trial in China found the seroconversion rates of EV71 antibody were higher than 94% in the infants who received the EV71 vaccine administered simultaneously with four Chinese Expanded Programme on Immunization(EPI) vaccines (hepatitis B vaccine, group A meningococcal polysaccharide vaccine, measles-rubella vaccine, and Japanese encephalitis vaccine), which were non-inferior to the EV71 vaccine separate administration group (97.93%) (19). The results of additional single-center phase 4 trials (20–22) also showed that the coadministration of EV71 vaccine with EPI vaccines did not interfere with the immunogenicity and increase the risk of AEs. As a result, the findings of different trial designs, vaccine manufacturers, and vaccines types administered simultaneously with EV71 vaccine were similar to our results, which supported the high seroconversion rates against EV71 (>94%) and good safety in the simultaneous administration groups and the EV71 vaccine group.

In this trial, we found that the GMT of EV71 antibodies in the EV71+IIV3 group was slightly lower than that in the EV71 group (419.05 vs 503.72) on day 56. Considering the non-inferiority was met for the GMT and seroconversion rate of EV71 neutralizing antibodies [GMT ratio: 0.84 (95%CI: 0.72-0.97); seroconversion rates difference: 0.02% (95% CI: –1.77-1.80)], and there was no difference in the seroconversion rate and seropositive rate of EV71 neutralizing antibodies on day 56 between the EV71+IIV3 group and EV71 group, thus further studies are needed to verify whether coadministration of EV71 vaccine with IIV3 would slightly affect the EV71 antibody response.

Our trial has some limitations. Infants under the age of 2-3 years were not enrolled, due to the busy immunization schedule during the first year of life. Another limitation is that we conducted this trial with IIV3. Given that only IIV3 was approved for use in children under 3 years of age in China at the start of our study, future studies on the coadministration of EV71 vaccine with a quadrivalent influenza vaccine are needed.

In conclusion, the coadministration of EV71 vaccine with IIV3 was safe with satisfactory immunogenicity against both EV71 and influenza virus. In the context of the COVID-19 pandemic and influenza season, the coadministration of EV71 vaccine and IIV3 is feasible, which could reduce the cost of vaccination and contribute to epidemic control in children.

## Data availability statement

The raw data supporting the conclusions of this article will be made available by the authors, without undue reservation.

## Ethics statement

The studies involving human participants were reviewed and approved by the ethical review committee of Zhejiang provincial center for disease control and prevention (CDC), Henan provincial CDC and Guizhou provincial CDC. Written informed consent to participate in this study was provided by the participants’ legal guardian/next of kin.

## Author contributions

ZC and XY contributed to the concept and design. YC, YY, FJ, HH, LS, QM, JL and QG were responsible for data collection and curation. YX, LL, HC, WC, XG and MZ were responsible for study supervision. YC, YY and FJ contributed to the formal analyses. YC and YX drafted the manuscript. ZC and XY were responsible for reviewing and editing the manuscript. All authors contributed to the article and approved the submitted version.
