# Supplementary material for: Immunogenicity and safety of an inactivated enterovirus 71 vaccine coadministered with trivalent split-virion inactivated influenza vaccine: A phase 4, multicenter, randomized, controlled trial in China
Source: Front Immunol. 2022 Dec 8;13:1080408. doi: 10.3389/fimmu.2022.1080408 (PMC9772018; doi:10.3389/fimmu.2022.1080408)
Supplement: Supplementary file 1 [file Table_1.docx]

Supplementary Material

Immunogenicity and safety of an inactivated enterovirus 71 vaccine coadministered with trivalent split-virion inactivated influenza vaccine: A phase 4, multicenter, randomized, controlled trial in China

Yaping Chen, Yanhui Xiao, Ying Ye, Feng Jiang, Hanqing He, Linyun Luo, Haiping Chen, Lubin Shi, Qiuyue Mu, Wei Chen, Xue Guo, Min Zhang, Jun Li, Qinghu Guan, Zhiping Chen*, Xiaoming Yang*

*** Correspondence:** Xiaoming Yang: [yangxiaoming@sinopharm.com](mailto:yangxiaoming@sinopharm.com)

Zhiping Chen: zpchen1963@163.com

# Supplementary Methods

**Method for haemagglutination inhibition test**

The influenza virus antibody titer was detected using a haemagglutination inhibition (HI) test. Serum was separated and stored at −20°C until tested by the Chinese National Institute for Food and Drug Control. The protocol of HI testing to measure antibody responses was briefly described. The serum samples were melted at room temperature, then cholera filtrate was added, incubated in a 37 °C incubator for 18 hours, inactivated in a 56°C water bath for 50 minutes. After the serum sample temperature reached room temperature, 50% chicken red blood cells were added, mixed thoroughly, and kept at 4°C for the night. Serum samples were diluted 1:5 and diluted 2-fold 11 times serially. 4 units of hemagglutinin antigen were added, vortexed and mixed, then kept for 45 minutes. 1% chicken red blood cells were added and mixed, and kept for 45 minutes. The highest serum dilution of completely inhibiting erythrocyte agglutination was used as the HI titer.

# Supplementary Tables

Table S1. Reported vaccine-related adverse events following any vaccination within 7 days.

| **Adverse events** | EV71+IIV3 group  (n=378) | EV71 group  (n=378) | IIV3 group  (n=378) | P |
| --- | --- | --- | --- | --- |
| **Total, n (%)** | 94(24.87) | 77(20.37) | 85(22.49) | 0.335 |
| **Systemic, n (%)** | 91(24.07) | 75(19.84) | 82(21.69) | 0.369 |
| Fever, n (%) | 65(17.20) | 56(14.81) | 49(12.96) | 0.263 |
| Rash, n (%) | 11(2.91) | 8(2.11) | 6(1.59) | 0.460 |
| Diarrhea, n (%) | 5(1.32) | 2(0.52) | 8(2.12) | 0.161 |
| Vomiting, n (%) | 2(0.53) | 0(0) | 0(0) | 0.333 |
| Decreased appetite, n (%) | 1(0.26) | 0(0) | 0(0) | 1.000 |
| Irritability, n (%) | 0(0) | 0(0) | 1(0.26) | 1.000 |
| Drowsiness, n (%) | 1(0.26) | 0(0) | 0(0) | 1.000 |
| **Local, n (%)** | 5(1.32) | 3(0.79) | 6(1.59) | 0.603 |
| Redness, n (%) | 0(0) | 2(0.53) | 0(0) | 0.333 |
| Induration, n (%) | 4(1.06) | 2(0.53) | 5(1.32) | 0.526 |
